# Genetic causes of intellectual disability in a birth cohort: A population‐based study

**DOI:** 10.1002/ajmg.a.37011

**Published:** 2015-02-27

**Authors:** Simone M. Karam, Mariluce Riegel, Sandra L. Segal, Têmis M. Félix, Aluísio J. D. Barros, Iná S. Santos, Alicia Matijasevich, Roberto Giugliani, Maureen Black

**Affiliations:** ^1^ Programa de Pós‐Graduação em Saúde da Criança e do AdolescenteUFRGSPorto AlegreBrazil; ^2^ Faculdade de MedicinaUniversidade Federal do Rio Grande (FURG)Rio GrandeBrazil; ^3^ Serviço de Genética MédicaHCPAPorto AlegreBrazil; ^4^ Programa de Pós‐Graduação em Genética e Biologia MolecularUFRGSPorto AlegreBrazil; ^5^ Programa de Pós‐Graduação em EpidemiologiaUFPelPelotasBrazil; ^6^ Department of Preventive MedicineSchool of MedicineUniversity of São PauloSão PauloBrazil; ^7^ Departamento de GenéticaUFRGSPorto AlegreBrazil; ^8^ John A Scholl MD and Mary Louise Scholl MD Endowed Professor, Department of Pediatrics and Department of Epidemiology and Public HealthUniversity of MarylandBaltimoreMaryland

**Keywords:** intellectual disability, mental retardation, genetics causes of diseases, population‐based studies, birth defects

## Abstract

Intellectual disability affects approximately 1–3% of the population and can be caused by genetic and environmental factors. Although many studies have investigated the etiology of intellectual disability in different populations, few studies have been performed in middle‐income countries. The present study estimated the prevalence of genetic causes related to intellectual disability in a cohort of children from a city in south Brazil who were followed from birth. Children who showed poor performance in development and intelligence tests at the ages of 2 and 4 were included. Out of 4,231 liveborns enrolled in the cohort, 214 children fulfilled the inclusion criteria. A diagnosis was established in approximately 90% of the children evaluated. Genetic causes were determined in 31 of the children and 19 cases remained unexplained even after extensive investigation. The overall prevalence of intellectual disability in this cohort due to genetic causes was 0.82%. Because this study was nested in a cohort, there were a large number of variables related to early childhood and the likelihood of information bias was minimized by collecting information with a short recall time. This study was not influenced by selection bias, allowing identification of intellectual disability and estimation of the prevalence of genetic causes in this population, thereby increasing the possibility of providing appropriate management and/or genetic counseling. © 2015 The Authors. *American Journal of Medical Genetics Part A* Published by Wiley Periodicals, Inc.

## INTRODUCTION

Approximately 3–10% of children are affected by some type of disability [Shevell et al., 2003], including cerebral palsy, epilepsy, blindness, autism, and intellectual disability (ID) [Shevell et al., 2003]. ID is a condition with medical, educational, and social importance [McDermott et al., 2007] and may be caused by genetic and environmental factors. ID affects about 2–3% of the general population [Curry et al., 1997; De Vries et al., 2005]. A varying proportion of ID cases (ranging from 17% to 50%) are attributable to genetic causes [Moeschler and Shevell, 2006; Kaufman et al., 2010].

Etiologic ID studies have generally been performed in selected samples [Butler and Singh, 1993; Félix et al., 1998; Santos et al., 2000] rather than population‐based cohorts [see El‐Hazmi et al., 2003; Lundvall et al., 2012; Gustavson, 2005 for exceptions]. Differences in sampling procedures may explain some of the variation in the proportion of cases due to genetic disorders.

Despite the fact that ID with or without congenital anomalies is the most frequent reason for seeking genetic advice [Rauch et al., 2006] and that it is considered the leading socio‐economic health care problem in Western countries, ID receives very little public attention [CDC, 2004; Salvador‐Carulla and Bertelli, 2008]. The need for initiatives to prevent disabilities, including ID, is pronounced in less developed countries. However, effective prevention requires better information on risk factors and causes [Durkin et al., 2000]. This study assessed the prevalence of ID due to genetic disorders in a population that was prospectively followed since birth and evaluated the etiology and associated factors related to ID in order to distinguish between genetic factors and factors linked to lifestyle, birth conditions, and socio‐economic status. At 7 years of age, children who had presented with developmental delay or suspected developmental delay by the ages of 2 and 4 years were evaluated by a clinical geneticist. The definition of ID was based on recommendations from the American Association on Intellectual and Developmental Disabilities (AAIDD) [Salvador‐Carrulla et al., 2011], and classification was based on ICD‐10‐WHO [WHO, 1996].

## MATERIALS AND METHODS

**Figure 1:**
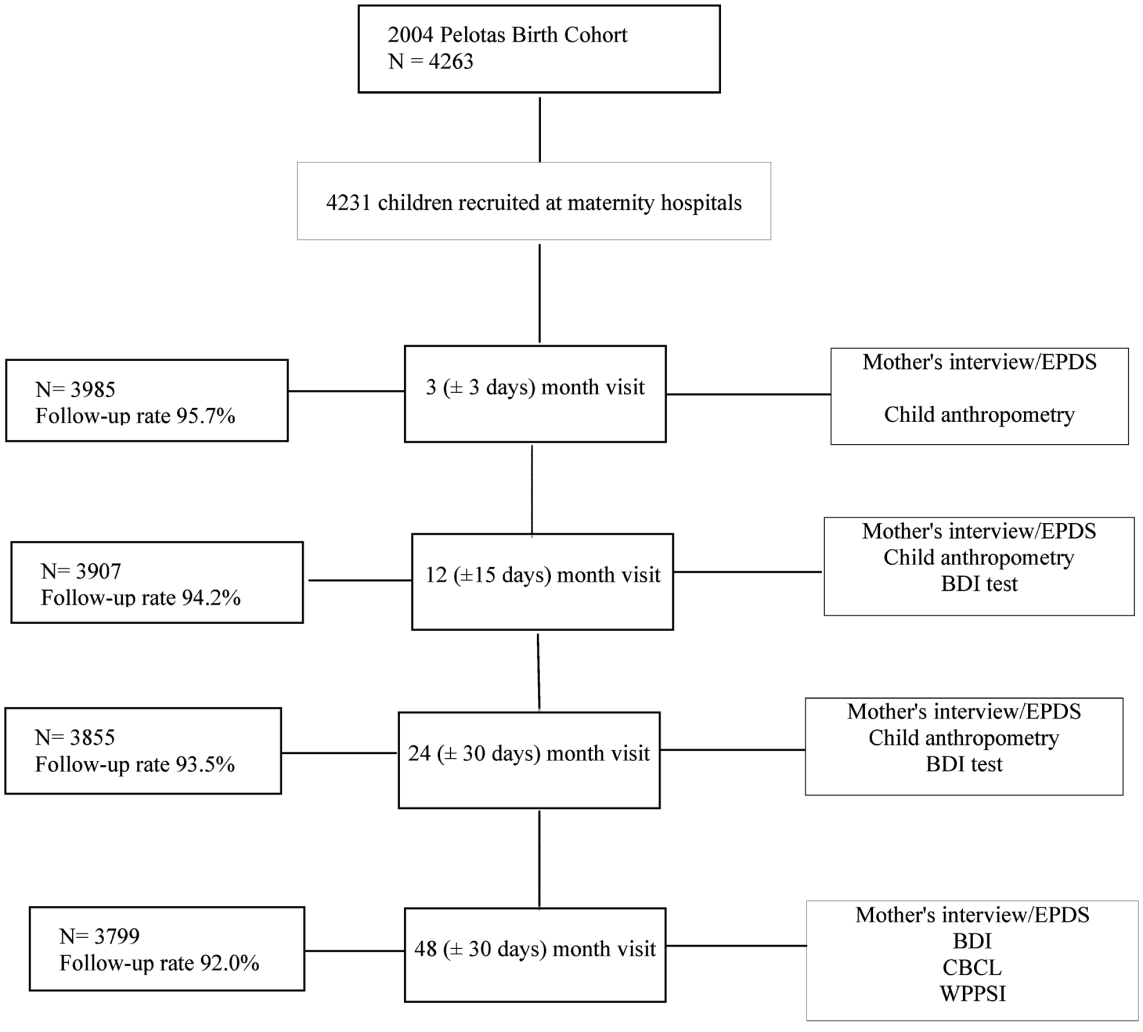
Flowchart outlining recruitment, visits, and procedures related to the 2004 Pelotas Birth Cohort study. EPDS, Edinburgh Postnatal Depression Scale; BDI, Battelle Development Inventory; CBCL, Child Behavior Checklist; WPPSI, Wechsler Preschool and Primary Scale of Intelligence; WISC, Wechsler Intelligence Scale for Children; CPT‐II, Conners' Continuous Performance Test; DAWBA, Development and Well‐Being Assessment for Children and Adolescents.

This study was nested in the 2004 Pelotas Birth Cohort Study. Pelotas is a city in southern Brazil with a subtropical and humid climate, a population of approximately 341,000 inhabitants, and an economy based on commerce and farming [IBGE, 2010]. The adult literacy rate is 95.7% [IBGE, 2010] and the infant mortality rate 16.3/1,000 [da Silva et al., 2012]. The majority of the population is white (80.3%), followed by black (10.7%), mixed race (8.6%), Asian (0.3%), and Native American (0.1%). Most of the original immigrants who settled in the city were Portuguese and German. Pelotas has not been growing at the same economic rate as other Brazilian cities [IBGE, 2010]. The per capita gross domestic product (GDP) was US$ 2,732 in 2002, which is lower than Brazil's average GDP of US$ 3,633 [IBGE, 2010]. From January 1st to December 31st 2004, all newborns were identified and their mothers were interviewed within 24 hr after delivery using a standardized pre‐coded questionnaire, which included the following nine sections: identification; delivery and child health; antenatal care and gestational morbidity; reproductive history; maternal characteristics and lifestyle; paternal work and family income; medical antenatal care tests; physical examination of newborn; and contact information. The newborn evaluation included length, cephalic, and abdominal circumference measurements [Barros et al., 2006]. Gestational age was assessed using the Dubowitz method [Dubowitz et al., 1970], the date of last menstrual period noted on the pregnancy card or as reported by the mother (in that order of priority) and ultrasound performed before 20 weeks of gestation. Birth weight was recorded from hospital nursing records. All hospitals used the same type of pediatric scale accurate to 10 g [Barros et al., 2006]. The scales were calibrated weekly by the staff using standard weights [Barros et al., 2006]. Maternal medical records were reviewed for information regarding prenatal care, maternal diseases, use of medicines, and previous pregnancies [Barros et al., 2006]. Non‐hospital deliveries (*n* = 20) were included, as most mothers are evaluated at a maternity ward soon after delivery [Barros et al., 2006; Barros et al., 2010]. In 2004, 4,558 children were born in Pelotas, including rural and urban areas and fetal deaths. Of the 4,263 live births whose families lived in the urban area and were eligible to enroll in the study, the mothers of 32 newborns refused to participate or were lost to follow‐up. As such, a total of 4,231 children were successfully recruited. Follow‐up evaluations were scheduled at 3, 12, 24, and 48 months of age and again at 6–7 years old (see Fig. 1). At each visit, the mothers were interviewed and evaluated for depression and general health. The children were examined, including a child development assessment. Assessments were conducted at the participants' home, except for the 6–7‐year‐old visit, which was conducted at a clinical research facility [Barros et al., 2006].

### Developmental Assessment

At 24 months, the screening version of Battelle Developmental Inventory (BDI) was administered. The BDI includes five domains: personal–social, adaptive, fine and gross motor, communication, and cognitive [Newborg et al., 1988]. BDI was translated to Portuguese from the Spanish version, followed by pre‐testing and revision to ensure cultural relevance. Interviewers were trained by a pediatric specialist in child development. Scores for each domain were summed to generate a total score [Newborg et al., 1988]. At 48 months of age, an abbreviated version of the Wechsler Preschool and Primary Scale of Intelligence test (WPPSI) including the Arithmetic, Block Design‐Picture, Pictures Completion, and Similarities [Kaufman, 1972; Wechsler et al., [Link]] was administered by trained psychologists.

### Risk of ID

Children were identified as being at risk of ID using the following criteria:

‐ BDI screening test for developmental problems  < −1 standard deviation (SD) at 24 months of age. Individual results were classified as “normal” or “suspected delay” according to a cut‐off point of −1 SD of the total reference population scores [Moura et al., 2010, 2010].
‐ WPPSI estimated IQ < 70 points at 4 years of age.
‐ One or more the following problems noted by interviewer at the end of the standardized questionnaire as the reason for not performing an IQ test at the 4 year old visit: communication difficulty (comprehension/language); aggressive behavior, no interaction with interviewer; or inability or refusal to complete the WPPSI test.

Children who met at least two of the criteria (*n* = 170) were invited to participate in a genetic evaluation at the research clinic at the age of 7–8 years. Parents or legal guardians signed informed consent for the collection of biological material and photographic records, and the child's cohort number was used as identification on all tests.

Forty‐four children were identified as being at risk for ID based on low scores from the BDI only without any other criteria. These children presented at birth with Apgar scores ≤6 and low birth weight or gestational age < 37 weeks. Despite these risk factors, they did not have dysmorphisms, behavioral, or adaptive problems and their IQs were higher than 70 based on WPPSI at age 4 and WISC at age 7. This subgroup was excluded from analysis.

### Genetic Evaluation

**Figure 2:**
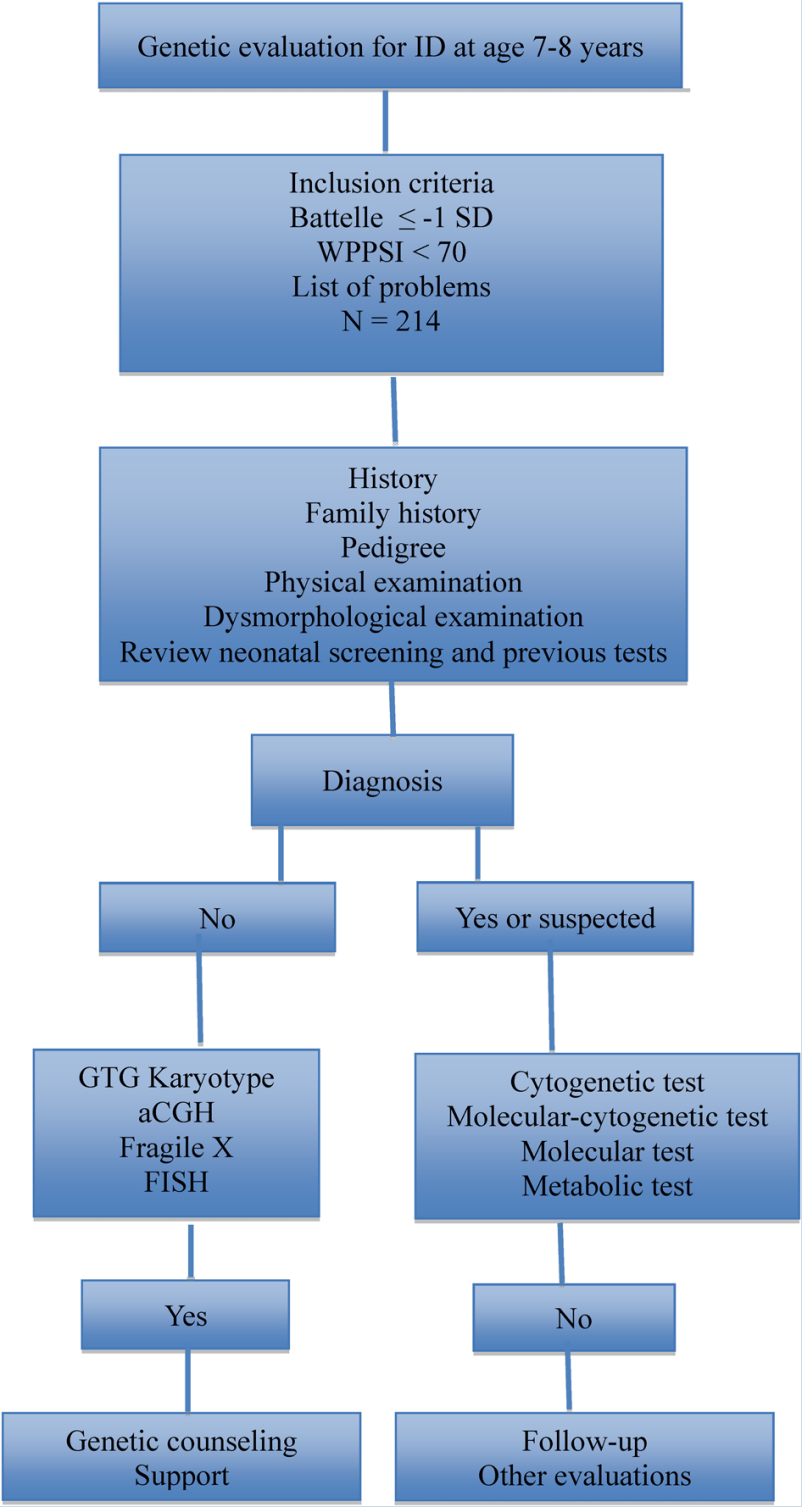
Flowchart outlining ID assessment in the 2004 Pelotas Birth Cohort study.

Genetic evaluation was conducted in a child‐friendly environment by a clinical geneticist. The evaluation consisted of an interview, pedigree of at least three generations and physical and dysmorphological examination (Fig. 2), including height, weight, cephalic circumference, and other measurements plotted as centiles. Adaptive behavior was assessed through maternal information and child observation. Mothers answered questions based on the Vineland‐II scale [Sparrow et al., 2005] concerning communication, daily living, and social skills. Children were assessed via observation and activities, respecting limitations such as vision impairments, as described in Table I. According to the diagnostic hypothesis, blood and/or urine samples were collected for the following tests:

**Table I:** Standard Protocol for Observation of Adaptive Behavior Concerning Communication, Daily Living, and Social Skills

Child observation						
Recognized						
Colors						
0	()	1–2	()	3 or more	()	
Numbers						
0	()	1–2	()	3 or more	()	
Alphabet letters						
0	()	2	()	More than 2	()	
Counted using his/her own fingers or objects						
Drew						
() Scribbles						
() A human form (head and body without members)						
() Complete human form						
() A landscape with several details						
Wrote his/her own name					() Yes	() No
Wrote simple words					() Yes	() No
Wrote sentences					() Yes	() No
Read						
Words						
0	()	1–2	()	3 or more	()	
Sentences (with 3 or more words)					() Yes	() No
A simple story (book)					() Yes	() No
Talked to investigator					() Yes	() No

#### Chromosome analysis

Metaphase chromosome preparations were obtained from phytohemaglutinin (PHA)‐stimulated lymphocyte cultures obtained from patients according to standard procedures. Chromosome analysis was performed using GTG banding at a 500–600 band level. A minimum of 20 metaphase chromosomes from each patient were examined. This test was requested when a child presented with possible ID associated with minor or major anomalies or a characteristic phenotype such as Down syndrome. A total of 47 karyotypes were analyzed.

#### Fluorescent in situ hybridization (FISH)

FISH was requested in five cases with clinical suspicion of specific chromosomal microdeletions in which the previous karyotype was normal. Experiments were performed using standard techniques with commercially available locus‐specific probes (Cytocell®, Cambridge). Hybridizations were analyzed under a Zeiss Axioplan epifluorescence microscope. Subsequent analysis was performed by image acquisition using a CCD camera and digital image analysis. At least 30 cells were analyzed per hybridization. A chromosome region was considered normal when the FISH signal from the corresponding probe was present on both homologous chromosomes.

#### Array comparative genomic hybridization (aCGH)

Molecular cytogenetic investigations were performed in suspected patients of microdeletion/microduplication syndromes or when the GTG karyotype was normal, but structural chromosomal disorders could not be excluded as a possible diagnosis. Array‐CGH whole genome analysis using customized Agilent 60‐mer oligonucleotide‐based microarrays with a theoretical resolution of 40 kb (8 × 60 K, Agilent Technologies Inc., Santa Clara, CA) was used. Labeling and hybridization were performed according to the protocols provided by Agilent. Arrays were analyzed with a microarray scanner (G2600D) and Feature Extraction software (v9.5.1) (both from Agilent Technologies). Image analyses were performed using Agilent Genomic Workbench Lite Edition 6.5.0.18 with the statistical algorithm ADM‐2, and sensitivity threshold 6.0. Map position was based on the UCSC Genome Browser, February, 2009, gh19 (NCBI Build GRCH37‐reference sequence). A total of 31 tests were performed.

#### Polymerase chain reaction (PCR) for fragile X syndrome

Molecular examination for fragile X syndrome was performed for all males who had any of the following clinical features: prominent ears, elongated face, poor eye contact, hyperextensibility, or macroorchidism [Jones, 2007]. Considering that some of these characteristics may not be present at 7–8 years of age, the test was performed for males with non‐specific ID after the first clinical and laboratory evaluations (i.e., males who underwent clinical examination and GTG karyotype analysis and persisted with unexplained ID). Screening was performed according to O'Connel et al. [2002] in which the region containing CGG repeats plus 222 bp of flanking sequences are amplified. Amplicons were checked by agarose mini‐gel detection followed by ethidium bromide staining. If the results showed a negative reaction (no amplification), a second strategy consisting of multiplex PCR (two pairs of primers) was used to amplify the regions containing the CGG (FRAXA allele) and GCC repeats (FRAXE allele; internal control) according to the protocol described by Wang et al. [1995]. A total of 18 tests were performed.

#### Biochemical tests

Screening for inborn errors of metabolism was requested in suspected cases [García‐Carzola et al., 2006]. Available tests were as follows: screening tests for inborn errors of metabolism in urine; gas chromatography for organic acids in a random morning sample (10–20 ml); high performance liquid chromatography (HPLC) of amino acids in plasma and/or urine; transferrin electrophoresis in plasma; and acylcarnitine profiles (liquid chromatography associated with tandem mass spectrometry). In this study, a total of six tests were performed. In two cases, organic acids were measured because the children presented with failure to thrive, recurrent infections, previous hypotonia, and vomiting episodes. Transferrin electrophoresis was investigated for a child who presented with seizures, autistic behavior, and inverted nipples. Two children presented with epilepsy resistant to treatment and one had neurologic regression. All six children presented with developmental delay.

Children with genetic disorders were followed up after laboratory confirmation for genetic counseling, management and referral to other specialists. Inconclusive cases were discussed with another experienced clinical geneticist (TF) and returned for an additional clinical evaluation, guidance and referral to other specialists when indicated. Children were seen for three or more visits. If affected relatives were suspected, children were referred for evaluation.

At the 6–7‐year‐old assessment of the 2004 cohort [Santos et al., in press], the Wechsler Intelligence Scale for Children (WISC‐III) [Wechsler, 1991] was administered using the same sub‐tests administered at 4 months of age (Arithmetic, Block Design‐Picture, Pictures Completion, and Similarities) [Wechsler et al., [Link]]. Children with Down syndrome did not complete the IQ test, and children with significant comprehension, vision, or motor impairments were excluded from WISC execution but not from genetic evaluation.

Based on clinical and laboratory evaluation, ID was classified into five etiologic groups:

‐ Genetic: children presenting with clinical and/or laboratory diagnosis of genetic disorders.
‐ Idiopathic: children presenting with syndromal characteristics, such as dysmorphism and abnormal behavior, who did not receive a conclusive, definitive diagnosis after clinical and laboratory evaluation.
‐ Neonatal sequelae: children who had a birth injury, neonatal hypoxia, hypoglycemia, intracranial bleeding, neonatal meningitis, or extended hospitalization during the neonatal period.
‐ Other diseases: normal delivery and first days of life, but subsequent diagnosis of epilepsy, visual or hearing impairment.
‐ Environmental causes: children with no evidence of genetic disorders, neonatal sequelae, syndromal features or any other disease, but who had low social economic status, lack of stimulation, and/or socio‐affective deprivation and poor school performance.

This paper focuses on the genetic and idiopathic groups. Results from analysis of the other groups will be published separately. This study was approved by the Institutional Ethics Committee, and all participants signed an informed consent.

In this study, we describe distribution of the cohort based on antenatal and birth conditions, family history of genetic disorders, maternal age at delivery, presence of co‐morbidities and several social and economic variables, such as skin color and family income. Analysis was performed using *Stata* 13.0.

## RESULTS

A total of 3,799 children were visited at the age of 4. The majorities were white (67.7%), followed by black (12.4%), mixed race (14.4%), and other (5.5%). Gender distribution was 52% male and 48% female. Socioeconomic level was evaluated using the economic classification of the Brazilian Association of Population Survey Companies (ABEP), in which the highest level is designated A and the lowest is E. This classification is based on the accumulation of material wealth and schooling of the household head. More than half were considered level C (53.2%), 23.3% were level B, 17.1% were level D, and 3.8% were level E. Only 2.6% were considered level A.

Of the 3,799 children, 214 were identified as being at risk for ID. A total of 195 were selected to participate in genetic evaluation at age 7–8, with 3 refusals, 16 children lost to follow‐up, and 44 were excluded because they did not have dysmorphic features, behavioral, or adaptive problems and their IQs were higher than 70 based on WPPSI at age 4 and WISC at age 7.

The remaining 151 children at risk for ID were classified into five groups based on genetic evaluation: environmental causes (*n* = 67); neonatal sequelae (*n* = 20); linked to other diseases (*n* = 14); idiopathic (*n* = 19); and genetic (*n* = 31).

The genetic group included 20.5% of the children at risk for ID. Almost 90% of mothers reported that their pregnancy was planned, all (100%) received prenatal care, and 70% had seven or more antenatal doctor visits. Twenty‐one percent of the mothers reported one or two previous miscarriages and 4% reported a diagnosis of gestational diabetes mellitus, although none reported being treated. Four of the seven mothers whose children were born with Down syndrome were 35 years or older at the time of delivery. Heart disease was the most common co‐morbidity in the genetic group. The main features of this group are shown in Table II. Almost 40% of the children had a diagnosis at birth, and more than half were diagnosed after the age of 4. Table III shows Battelle test scores that fell below normal standards and IQ according to WPPSI and WISC scales for children with genetic disorders, including chromosomal, Mendelian, or multifactorial inheritance. Chromosomal disorders were the main genetic cause of ID, with Down syndrome occurring most frequently, equivalent to 1:605 births. Chromosomal disorders were followed by autosomal dominant (AD) and multifactorial inheritance disorders (Table III). AD disorders were sporadic cases, except in a family with AD microcephaly.

**Table II:** Characteristics of Children With Intellectual Disability With a Genetic Etiology

	*N* (%)
Gender	
Male	16 (52.0)
Female	15 (48.0)
Gestational age (weeks)	
≤37	6 (19.4)
37–42	25 (80.6)
Birth weight	
<2,500 g	4 (12.9)
2,501–3,500 g	24 (77.4)
3,501–4,500 g	3 (9.7)
Apgar score	
0–6	0 (0.0)
7–10	31 (100.0)
Family history	
Yes	4 (12.9)
No	27 (87.1)
Maternal age (years)	
<19	4 (12.9)
20–30	14 (45.2)
30–35	7 (22.6)
≥36	6 (19.3)
Medical/surgical problems	
Visual impairment	4 (12.9)
Partial or total deafness	4 (12.9)
Seizures	2 (6.5)
Cardiopathy	7 (22.6)
Mobility difficulties	5 (16.1)
Age at diagnosis	
At birth	12 (38.7)
1–2 years	3 (9.7)
≥4 years	16 (51.6)

**Table III:** Battelle Test Scores Below Normal Standards and IQ Results According to WPPSI and WISC Scales Among Children With Genetic Disorders, Including Chromosomal, Mendelian, or Multifactorial Inheritance

Diagnosis	Sex	Battelle (SD)	WPPSI	WISC‐III
Chromosomal				
Williams syndrome	F	−1.5	57.52	E
Down syndrome	F	−1	57.52	E
Down syndrome	M	−1.5	62.41	E
Down syndrome	F	−1	60.78	E
Down syndrome	F	−2	57.52	E
Down syndrome	M	−2	64.04	E
Down syndrome	M	−2	57.52	E
Down syndrome	M	NS	57.52	E
Del 14q32.31–q32.33	F	−2	67.3	44
Del 2q22.3–q23.1 (394.162 pb)	F	−1.5	57.52	44
Gain 3q29 uncertain	M	NS	67.3	49
Gain22q11.23 uncertain	M	NS	72.19	68
Mendelian/sporadic				
Cornelia de Lange syndrome	F	Missing	57.52	E
Autossomal dominant microcephaly	F	NS	60.78	44
Fragile X syndrome	M	NS	60.78	42
Moebius sequence	M	−1.5	59.15	E
Noonan syndrome	M	−1	62.41	47
Noonan syndrome	F	NS	67.3	52
Tuberous sclerosis	M	−2	57.52	E
Multifactorial				
ADHD	F	−1	Missing	E
ADHD	M	NS	68.93	52
ADHD	F	NS	65.67	47
ADHD	M	NS	Refuse	E
ADHD	M	NS	64.04	57
ADHD	F	NS	65.67	57
Porencephalic cyst	F	−2	64.04	55
Schizencephaly	M	−2	57.52	E
Myelomeningocele	F	−2	81.97	63
Myelomeningocele	F	NS	68.93	52
Myelomeningocele	M	Missing	57.52	E
Myelomeningocele and hydrocephaly	M	−2	57.52	E

SD, standard deviation; NS, normal standard; E, excluded; M, male; F, female; ADHD, attention deficit hyperactivity disorder.

Based on the BDI test, which is used to screen for developmental delay at age 2, 17 children were delayed (55%), but at least three children from each group had a normal result. Based on the WPPSI scale at age 4, 27 of 31 children had an IQ below 70 points. ID level was classified as mild or moderate according to the IQ test. At age 7, all children with genetic disorders who were tested presented with an IQ below 70, although 16 children could not be classified because they did not complete the WISC‐III and were excluded from this test. Thirteen of the children attended special education schools at the time of the study and 18 were in general education mainstream schools.

The prevalence of genetic ID at the age of 7 was 0.82% based on the number of visits at the last Cohort assessment (3,799) and was the second most frequent cause of ID in this cohort.

We identified the following disorders (Table III): Cornelia de Lange, Noonan, Williams, Moebius, fragile X, and Down syndromes, tuberous sclerosis, autosomal dominant microcephaly (proband, mother, and two siblings), abnormal microarrays and multifactorial disorders such as schizencephaly, porencephalic cyst, and myelomeningocele. Most of the disorders were clinically diagnosed. The two patients with Noonan syndrome had requested GTG karyotyping to exclude other syndromes related to ID, short stature, and dysmorphic features. The child with Williams syndrome fulfilled the clinical criteria for the referred syndrome, and although the GTG karyotype was normal, FISH confirmed the diagnosis of a microdeletion at 7q11.23. The patient with fragile X syndrome fulfilled the clinical criteria for diagnosis, which led to molecular confirmation. Children who presented with aCHG abnormalities were extensively investigated. The first patient (del14q32.31–q32.33) had been seen at an outpatient clinic at the age of 2 and was characterized by global hypotonia, developmental delay and some dysmorphic features. GTG karyotype was normal and electromyogram and muscle enzymes were evaluated. Learning difficulties and behaviors, such as irritability and restlessness, were observed in a school setting. The second patient (del2q22.3–q23.1) was suspected based on clinical examination and similarities between the proband and her mother, suggesting a structural chromosomal alteration. The other two patients with an abnormal aCGH (3q29 gain and 22q11.23 gain) were both males and presented with a positive family history for ID and behavioral problems without dysmorphic features. One of the patients also presented with (3q29 gain) seizures. These cases were submitted for routine clinical and laboratory evaluations. PCR analysis for fragile X syndrome was performed before aCGH.

A total of 19 children (12.6%) were classified as having idiopathic ID (i.e., unable to clearly determine a diagnosis even after extensive clinical and laboratory investigations). Table IV shows the clinical characteristics of children with idiopathic ID and performance on screening development and intelligence tests. Children who presented with dysmorphism or adaptive behavioral impairments or autistic features (*n* = 15) were analyzed using GTG karyotyping and aCGH. PCR for fragile X was performed in males. For overweight patients, karyotype, aCGH and FISH for Prader–Willi syndrome were performed.

**Table IV:** Clinical Characteristics of Children With Intellectual Disability of Unknown Cause (Idiopathic ID) and Their Performance in Developmental Screening/IQ Tests (Lower and Upper Scores on Developmental Screening and Intelligence Tests)

Clinical features	*N*	%	BDI range	WPPSI	WISC
ID associated with dysmorphism and disturbances in adaptive behavior	7	36.8	NS/−1	57.52/78.47	49/63
ID without dysmorphism and associated with disturbances in adaptive behavior	5	26.4	NS/−1	57.52/85.23	44/54
ID associated with autism	3	15.8	NS/−2 SD	57.52/72.19	0/65
ID without dysmorphism associated with overweight	2	10.5	NS	54.0/57.52	42/76
ID associated with motor delay	2	10.5	−1/−1.5	81.97/83.6	0/54
Total	19	100			

SD, standard deviation; NS, normal standard; 0, did not take the test.

Although three mothers reported alcohol consumption during pregnancy, the children were not evaluated because they could not be located after several attempts and were considered lost to follow‐up. All 195 children underwent neonatal screening for congenital hypothyroidism, phenylketonuria, and hemoglobinopathies. There were no diagnoses of congenital hypothyroidism or inborn errors of metabolism, and there were no consanguineous marriages registered (data not shown).

## DISCUSSION

The prevalence of ID is highly variable depending on the country studied, the age of the subjects and the method of determination [Kaufman et al., 2010]. In high‐income countries, the prevalence is usually lower than in low and middle‐income countries. However, most of the studies are based on administrative data obtained using standardized diagnostic systems, which can exclude mild cases on a functional level, particularly when assessments are school‐based given that these cases are often classified as “learning disorders” [Maulik et al., 2011]. Some multi‐country studies [Stein et al., 1987] have reported a higher prevalence in low and middle‐income countries using the Ten Question (TQ) instrument, which is not specific for intellectual disabilities and does not include functional level. There are several important factors that may contribute to a higher prevalence in these countries, including birth‐related infections and injuries due to poor maternal and child health facilities, intrauterine growth restriction and a proportionately higher number of births with hereditary illnesses due to lack of adequate prenatal screening methods [Maulik et al., 2011]. In this study, we focused on prevalence of genetic causes of ID. The prevalence observed in this cohort provides an actual estimate because the rate was not obtained from select populations, such as people with disabilities who live in care homes, but rather from a population‐based study. In our study, about 20% of the cases of ID were attributable to genetic abnormalities, other studies have reported that genetic abnormalities are responsible for 15–50% of the cases of ID [Hunter, 2000; Chelly et al., 2006; Moeschler and Shevell, 2006; Gonzáles et al., 2013; Shashi et al., 2014].

Down syndrome represented the majority of chromosomal disorders, as reported in other Brazilian studies [Félix et al., 1998; Santos et al., 2000], and its frequency was similar to that reported in the literature [CDC, 2006; Sherman et al., 2007; Shin et al., 2009]. Down syndrome is the most common genetic cause of ID [Sherman et al., 2007; McDermott et al., 2007]. Chromosomal abnormalities are responsible for approximately 40% of severe ID cases and 10% of mild cases.

The frequency of neural tube defects (NTDs) was similar to that described in the literature, approximately one for every thousand live births [Grilo and Melo da Silva, 2003; McDermott et al., 2007], although higher rates have been reported in Brazil [Aguiar et al., 2003]. Children with NTDs have an increased risk for ID [Jelliffe‐Pawlowiski et al., 2003]. Fragile X syndrome, which is a common cause of ID [Guillén‐Navarro and Glóver‐Lopez, 2006; Raymond and Tarpey, 2006; Ropers and Hamel, 2005], was found in our sample population.

GTG karyotyping is the standard method for investigation [Clarkson et al., 2002] of chromosomal abnormalities. Array based genomic comparative hybridization is indicated for detecting structural chromosomal abnormalities between 3 and 5 Mb, which are considered too small to be diagnosed by conventional cytogenetic techniques. Using aCGH, a patient's genomic DNA and a control are co‐hybridized, determining gains and losses of certain chromosome segments [Shaw‐Smith et al., 2004], as were found in four cases in the present study. Although two abnormal microarrays were described as uncertain, we included them in Table III because other ID causes had been ruled out.

In cases of suspected classic microdeletion syndromes, our first choice was the FISH method. When the diagnosis remained undefined, aCGH was performed. The literature suggests that this technique is an important research tool when GTG banding and FISH results are normal and likely increases the chance of reaching a diagnosis [Schoumans et al., 2005; Stamkiewicz and Beaudet, 2007; Manolakos et al., 2010]. All of the children with cytogenetic disorders in this study had never undergone genetic testing, as the Public Health System does not routinely offer these tests. This study allowed a diagnosis and genetic counseling for most of the children identified as being at risk for ID in this cohort and provided additional information and future follow‐up for children with idiopathic ID. Patients were referred to other specialists, such as occupational therapists, ophthalmologists and ENT specialists. Despite technical advances in neurological, metabolic, cytogenetic and molecular testing, approximately 30% of severe ID cases and 70% of mild ID cases remain without a definitive diagnosis [Gonzáles et al., 2013]. Patients in the idiopathic ID group are candidates for future exome sequencing, which has been suggested as a diagnostic procedure for patients with severe ID of unknown cause. Mutations in more than 1,000 different genes may cause intellectual disability, and evidence supporting the hypothesis that rare de novo point mutations can be a major cause of ID has been reported [de Light et al., 2012]. Both microarray studies and exome sequencing have demonstrated the importance of de novo copy number variations (CNVs) and single‐nucleotide variations (SNVs) in ID as a possible causation [Glissen et al., 2014].

Children affected by ADHD are included in the group with genetic ID. ADHD has an estimated heritability of approximately 80% but its nosologic definition is controversial. Although a substantial fraction of its etiology may be genetic many environmental risk factors and potential gene‐environment interactions also increase the risk for the disorder [Das Banerjee et al., 2007] and cultural aspects should also be considered [Polancyk et al., 2007]. Recent studies have suggested a role for CNVs and candidate genes, which could explain the variability of phenotypic heterogeneity in ADHD and other psychiatric disorders [Elia et al., 2010]. Population studies have demonstrated that different genetic loci are related to a higher prevalence of autism spectrum disorder, dyslexia, and ADHD [Fernandez‐Jaén et al., 2014]. Similar to many other psychiatric disorders, ADHD does not have a classical mode of inheritance and causation is considered multifactorial.

Children whose clinical diagnosis or suspicion of ID occurred during first the 2 years of life primarily presented with Down syndrome. For most other cases, diagnostic suspicion occurred after 4 years or during this study. The phenotype of some genetic syndromes could evolve slowly [Daily et al., 2000], and subtle neurological anomalies and psychiatric disorders tend to be less apparent due to cognitive impairment [Kaufman et al., 2010], leading to a delayed diagnosis. In addition, new syndromes are constantly being reported [Daily et al., 2000], reinforcing the need for systematic follow up [Battaglia and Carey, 2003].

It is important to discuss the inclusion criteria. The first point to consider is that these children were recruited from an epidemiological study, and development and intelligence assessments were applied on a large scale with the objective of screening for these problems, not to diagnose a particular condition. The three criteria were selected to increase sensibility, identify more children at risk for ID and allow for clinical evaluation. Although developmental delay, which can be suggested by BDI and other screening tests, can be transient [Moura et al., 2010, 2010] when it is mild, this condition implies learning and adaptation deficits that could predict ID [Moeschler and Shevell, 2006].

We evaluated all children who presented with at least one of the criteria and decided to exclude 44 children with WPPSI and WISC scores ≥70 who did not have any behavior or adaptive problems after clinical evaluation.

There was a considerable decrease in IQ scores between the ages of 4 and 7. Everyone who completed the trial had a lower IQ score compared to previous assessments. The general rule is that IQ is more stable in older children, although genetics, familial, and educational factors can influence performance [Braaten and Norman, 2006]. However, for patients with fragile X [Fisch et al., 1996; Bennetto and Pennington, 1996] and trisomy 21, a decline in IQ is expected [Wishart, 1993] regardless of social class [Carr, 1995].

There are several limitations to the present study. We screened school age children, and it is possible that cases of mild ID could appear later in life. In addition, a limited number of laboratory tests were able to be performed. Continued follow‐up could provide additional valuable information, particularly regarding idiopathic disturbances and ADHD.

Given the lack of population‐based prevalence studies of ID in Brazil, particularly regarding genetic causes, this study allowed identification of ID and its etiology in approximately 90% of cases in this population using essential tools as recommended in the literature (detailed clinical history, physical examination, and basic laboratory investigations). Discovering the etiology of ID can provide useful information to individuals with ID and their families. This study could contribute to developing future projects on this topic and/or to planning strategies that identify ID early in an effort to improve access to management interventions and specialized care.
